# Infection control, hand hygiene practice and PPE use among phoniatricians and ENT specialists during the COVID-19 pandemic, a UEP survey

**DOI:** 10.1186/s43163-022-00205-1

**Published:** 2022-01-24

**Authors:** Mariam S. Shadi, Ahmed Geneid, John S. Rubin, Reham Abdelwakil Ibrahim

**Affiliations:** 1 Department of Otorhinolaryngology, Phoniatrics Unit, Faculty of Medicine, Ain Shams University, Abbassia, Cairo, 11566 Egypt; 2 Department of Otorhinolaryngology and Phoniatrics - Head and Neck Surgery, Helsinki University Hospital and University of Helsinki, Helsinki, Finland; 3 University College Hospitals London NHS Trust, London, UK; 4 National Hospital for Neurology and Neurosurgery, London, UK; 5 Department of Targeted Intervention, University College London, London, UK; 6 Department of Health Science, City University of London, London, UK; 7 Department of Otorhinolaryngology, Phoniatrics Unit, Assuit University, Assuit, Egypt

**Keywords:** Phoniatricians, Otolaryngologists, Infection control, Hand hygiene, PPE, COVID-19

## Abstract

**Background:**

Caring for our patients while taking care of our own safety as well as theirs is a major concern during the current pandemic. Therefore, many societies developed guidance documents to educate clinicians about the required precautions. This study aims to assess personal protective equipment (PPE) usage, hand hygiene practice and infection control training among phoniatricians and otolaryngologists during the pandemic. An online survey was administered during the first wave of the COVID-19 pandemic in June 2020. Data collected included PPE availability, infection control training, adopted infection control precautions, hand hygiene practice, and use of different PPE elements as well as adherence to its use during potential aerosol generating procedures.

**Results:**

Based on their country of residences, eligible 154 participants were grouped into 4 groups and their responses were compared.

**Conclusion:**

Following the suggested recommendations, while adequate for some precautions, was still not satisfactory. Certain defects that are specific to particular groups had also been identified.

## Background

Phoniatricians and otolaryngologists are at an increased occupational risk of COVID-19 infection [1–4] as they manage patients with diseases of the aerodigestive tract and evidence indicated that SARS-CoV2 viral density is greatest in the nose/nasopharynx of infected persons [2, 5]. Recommendations and guidance reports were therefore available to direct clinicians in implementing infection control measures and ensure their safety during the pandemic period [2, 5–15].

Standard infection control precautions (SICPs) should be enough for routine care of patients known to be negative [5]. Additional transmission-based precautions (TBPs) must be followed when managing patients with suspected or confirmed COVID-19 or if test results are pending [5, 6]; with ‘contact and droplet precautions during routine patient care’ and ‘contact, droplet and airborne precautions during aerosol generating procedures (AGPs)’ [5, 6].

Several medical procedures are identified as potentially AGPs [2, 5, 7, 8]. AGPs result in the release of airborne viral particles, either by mechanically dispersing aerosols or by inducing the patient to cough, gag or sneeze [1, 5, 7, 9]. This imposes a risk of airborne transmissions of infections that classically spread via droplet transmission [2, 5]. Any decision to perform these procedures should include consideration of the patient’s medical condition, COVID-19 status, risk and benefit analysis and personal protective equipment (PPE) availability [5, 7].

PPE generally describes equipment worn to reduce transmission of infectious particles and is an essential element of controlling COVID-19 infection [7, 9]. In the context of the current pandemic, it particularly refers to face coverings, eye, hand and body protections.

Determining the adequate PPE level depends on the patient’s COVID-19 status, the assumed duration of exposure and likelihood of aerosolization, PPE availability, and local regulations [6, 7]. Unsurprisingly, recommendations tend to advocate maximizing safety and taking a conservative approach, given the high rates of false negative test results [7], and the presence of asymptomatic carriers who may transmit the infection [7, 8]. The suggested minimum PPE requirements include a fit-tested N95 mask or Powered Air Purifying Respirator (PAPR) (a surgical mask only in case of performing non-AGPs on patients with an unknown or a negative COVID-19 status), disposable single-use gloves, fluid resistant eye protection e.g., face shield, visor, safety glasses, or goggles (and a gown in case of performing AGPs in COVID-19 positive patients) [7].

Special attention to infection control precautions is essential to minimize the risk of infection transmission. This survey was conducted during the first wave of the COVID-19 pandemic aiming to assess PPE use, hand hygiene practice and infection control training among phoniatricians and otolaryngologists. With the pandemic still far from over for many nations worldwide, we are planning to carry out a follow up survey to analyze how our practice had evolved over time.

## Methods

Online data collection method was adopted using a convenience sampling technique. A “Google form” was created, and the questionnaire link was shared with participants through UEP and other networks in the period between June 9th to 24th, 2020. Participants were aware of the study purpose and their data remained anonymous.

Survey questions involved inquiries into participants’ demographic data, availability of PPE, educational training related to infection control, type of infection prevention precautions followed, participants’ hand hygiene practice and their use of different PPE elements on dealing with patients. Participants were required to respond to each question by choosing one or more response(s), that describe their practice, from the provided list of options.

Preliminary pilot pretesting of the questionnaire involving 10 respondents took place, with subsequent slight modifications of questions following their feedback on relevance of content, time taken to complete, and clarity and coherence of questions. These 10 responses were not considered in data collection and statistical analysis.

### Statistical analysis

The collected data were coded, tabulated, and statistically analyzed using IBM SPSS statistics software version 22.0, IBM Corp., Chicago, USA, 2013. Descriptive statistics were done as Mean ± SD (standard deviation) for quantitative data and as number and percentage for qualitative data. For inferential analyses, ANOVA test was used for quantitative data differences, Chi square test for differences between proportions and Fisher’s Exact test for variables with small, expected numbers. The level of significance was set at *P* value < 0.050.

## Results

Among 202 respondents to the survey, 46 responses (for those working from home and not attending their workplace), in addition to 1 incomplete response and 1 non- eligible response were excluded. The remaining 154 responses were eligible and statistically analyzed.

Included responses were categorized according to the country of their residence into 4 groups; residents of Western Europe (*n* = 62) represent Group 1, with the largest number of responses from Germany (*n* = 30), residents of the Middle East (*n* = 37) with almost all responses from Egypt (*n* = 33) represent Group 2, Latin America (*n* = 36), predominantly Brazil (*n* = 33) in Group 3, and Eastern Europe (*n* = 15) in Group 4. A few responses (*n* = 4) also came from other countries.

### Demographic data

The mean age ± SD of the whole group of participants was 47 ± 11.8 years. The mean age of different groups (Table 1) showed statistically significant but not contextually significant differences, as all groups’ mean ages lie within the range of ‘middle age’. Females represented the majority (55.8%, *n* = 86) of participants, with 42.2% (*n* = 65) males and 1.9% (*n* = 3) chose not to specify their gender. Most participants have over 10 years of experience with no significant differences identified between groups. The majority of respondents within the entire group of participants (62.3%, *n* = 96) as well as within G1 (80.6%, *n* = 50), G2 (89.2%, *n* = 33) and G4 (46.7%, *n* = 7) were phoniatricians, while the majority in G3 (83.3%, *n* = 30) were laryngologists.

**Table 1:** Participants’ age and availability of PPE in their health care facilities

		All sample (***N*** = 154)	G1 (***N*** = 62)	G2 (***N*** = 37)	G3 (***N*** = 36)	G4 (***N*** = 15)	***P***-value
**Age (years)**	**Mean ± SD**	47.2 ± 11.8	51.4 ± 10.6	41.5 ± 11.0	47.3 ± 12.8	43.3 ± 9.2	^< 0.001*
**PPE availability:**	Yes	119 (77.3%)	50 (80.6%)	19 (51.4%)	34 (94.4%)	13 (86.7%)	#< 0.001*
	No	33 (21.4%)	11 (17.7%)	17 (45.9%)	2 (5.6%)	2 (13.3%)	
	I do not know	2 (1.3%)	1 (1.6%)	1 (2.7%)	0 (0.0%)	0 (0.0%)	

G1 (Western European), G2 (Middle Eastern), G3 (Latin American), G4 (Eastern European) countries’ participants

^ANOVA test #Chi square test. *Significant, SD standard deviation

### Availability of PPE

The majority of the total participants (*n*=119, 77.3%) reported that all PPE and protective measures they needed had been always available for them. That said, differences between groups were detected (see Table 1). Of note, most participants (*n*=110, 71.4%) have not had to perform AGPs without putting on the required level of PPE, while one fifth of participants (*n*=31, 20.1%) had, and 13 (8.4%) were not sure. No significant differences between groups were detected.

### Infection control educational training

The majority of participants (*n*=102, 66.2%) had been recently educated or had received a refresher training on how to put on (don), use, take off (doff), properly dispose PPE or disinfect and maintain reusable ones without self-contamination, and a statistically significant difference between groups is noticed (Table 2). Almost half (*n*=79, 51.3%) had received training in how to disinfect tools and surrounds. Only 66 (42. 9%) were trained in remedial actions against occupational exposure to COVID-19 e.g., how to deal with skin, mucus membrane or respiratory tract exposure, and a statistically significant difference between groups is observed (see Table 2). Furthermore, 36 (23.4%) had not received any training at all.

**Table 2:** Infection control training and precautions undertaken by participants

		All sample (***N***=154)	G1 (***N***=62)	G2 (***N***=37)	G3 (***N***=36)	G4 (***N***=15)	***P***-value
**Infection control training:**	PPE donning and doffing	102 (66.2%)	47 (75.8%)	17 (45.9%)	26 (72.2%)	9 (60.0%)	#0.017*
	Remedial actions against exposure to COVID-19	66 (42.9%)	26 (41.9%)	10 (27.0%)	22 (61.1%)	6 (40.0%)	
							#0.033*
**Type of infection prevention precautions followed:**	Droplet Precautions	28 (18.2%)	9 (14.5%)	6 (16.2%)	9 (25.0%)	2 (13.3%)	#0.015*
	Airborne Precautions	24 (15.6%)	12 (19.4%)	0 (0.0%)	8 (22.2%)	4 (26.7%)	
	Droplet and sometimes airborne Precautions	66 (42.9%)	32 (51.6%)	16 (43.2%)	13 (36.1%)	4 (26.7%)	
	Standard Precautions	36 (23.4%)	9 (14.5%)	15 (40.5%)	6 (16.7%)	5 (33.3%)	

G1 (Western European), G2 (Middle Eastern), G3 (Latin American), G4 (Eastern European) countries’ participants

#Chi-square test. *Significant

### Infection control precautions adopted

The majority of participants (*n*=127, 82.5%) empirically adhered to TBPs; which assume that every person is potentially infected, while 20 (13.0%) followed these extra precautions only with confirmed or suspected COVID-19 patients and 7 (4.5%) did not follow any extra precautions at all. No significant differences between groups are found.

In addition to SICPs, the largest number of participants (*n*=66, 42.9%) followed droplet precautions most of the times and shifted to airborne precautions during certain procedures, while 28 (18.2%) followed droplet precautions at all times and 24 (15.6%) followed airway precautions at all times. Nevertheless, 36 (23.4%) only followed standard precautions, nothing more. The statistically significant differences between groups are shown in Table 2.

### Equipment disinfection

In terms of taking extra care disinfecting the equipment shared by patients, the largest number of participants (*n*=111, 72.1%) always did so, 27 (17.5%) did so sometimes and 16 (10.4%) never did so. No statistically significant differences are noticed between groups.

### Hand hygiene (HH) practice

The majority of participants practiced HH before all patient contact (*n*=133, 86.4%) {no more than 9 (5.8%) disinfected hands only before aseptic procedures}, after all patient contact (*n*=131, 85.1%), after exposure to body fluid or any potentially infectious material (*n*=121,78.6%), after touching patient’s surroundings (e.g. bed, door handle) (104, 67.5%) and after touching or adjusting the mask (*n*=83, 53.9%). A smaller number of participants practiced HH before touching or adjusting their masks (*n*=74, 48.1%), before removing PPE (*n*=69, 44.8%), before putting on PPE (*n*=35, 22.7%), and 3 (1.9%) were not performing HH in any of these conditions. The significant differences detected between groups are shown in Table 3.

**Table 3:** Hand hygiene practice in the different groups of participants

	All sample (***N***=154)	G1 (***N***=62)	G2 (***N***=37)	G3 (***N***=36)	G4 (***N***=15)	***P***-value
**Hand hygiene before all patient contact:**	133 (86.4%)	56 (90.3%)	25 (67.6%)	36 (100.0)	13 (86.7%)	§< 0.001*
**Hand hygiene after exposure to infectious materials:**	121 (78.6%)	49 (79.0%)	31 (83.8%)	30 (83.3%)	7 (46.7%)	#0.019*
**Hand hygiene before touching or adjusting the mask:**	74 (48.1%)	29 (46.8%)	20 (54.1%)	20 (55.6%)	2 (13.3%)	#0.035*
**Hand hygiene after touching or adjusting the mask:**	83 (53.9%)	34 (54.8%)	19 (51.4%)	23 (63.9%)	3 (20.0%)	#0.039*

G1 (Western European), G2 (Middle Eastern), G3 (Latin American), G4 (Eastern European) countries’ participants

# Chi-square test. §Fisher’s Exact test. *Significant

### Use of different PPE elements while dealing with patients

#### Masks

The largest number of participants (*n*=69, 44.8%) always used an N95/N98 mask (or equivalent FFP2/FFP3 respirator in the EU) or a PAPR respirator, less (*n*=34, 22.1%) usually used a surgical mask, and an N95/N98 or a PAPR only during AGPs, or always used a standard surgical mask and think it is sufficiently protective (*n*=32, 20.8%), the remaining (*n*=19, 12.3%) used either masks according to their availability, and none of the participants refrained from using masks. Statistically significant differences between groups are shown in Table 4.

**Table 4:** Use of personal protective equipment (PPE) by the study groups on dealing with patients during the pandemic

Personal protective equipment (PPE) use		All sample (***N***=154)	G1 (***N***=62)	G2 (***N***=37)	G3 (***N***=36)	G4 (***N***=15)	***P***-value
**Masks/ respirators use:**	N95/N98 mask or PAPR	69 (44.8%)	32 (51.6%)	3 (8.1%)	28 (77.8%)	4 (26.7%)	§< 0.001*
	Surgical mask	32 (20.8%)	5 (8.1%)	20 (54.1%)	3 (8.3%)	4 (26.7%)	
	Surgical or N95/N98 mask based on availability	19 (12.3%)	8 (12.9%)	7 (18.9%)	3 (8.3%)	1 (6.7%)	
	Surgical mask, and an N95/N98 only during AGPs	34 (22.1%)	17 (27.4%)	7 (18.9%)	2 (5.6%)	6 (40.0%)	
	No mask use	0 (0.0%)	0 (0.0%)	0 (0.0%)	0 (0.0%)	0 (0.0%)	
**Mask replacement:**	Immediately after dealing with a patient	22 (14.3%)	6 (9.7%)	9 (24.3%)	2 (5.6%)	5 (33.3%)	§0.028*
	“Extended use” and change only if soiled or damp	115 (74.7%)	45 (72.6%)	26 (70.3%)	30 (83.3%)	10 (66.7%)	
	“Extended use” for the day even if soiled/damp	17 (11.0%)	11 (17.7%)	2 (5.4%)	4 (11.1%)	0 (0.0%)	
**Eye protection:**	Safety glasses with or without side protections	24 (15.6%)	11 (17.7%)	2 (5.4%)	6 (16.7%)	5 (33.3%)	§0.014*
	Occlusive eyewear e.g. swim goggles	13 (8.4%)	5 (8.1%)	1 (2.7%)	7 (19.4%)	0 (0.0%)	
	Face shield/visor	89 (57.8%)	36 (58.1%)	26 (70.3%)	16 (44.4%)	7 (46.7%)	
	Corrective/medical glasses only	18 (11.7%)	8 (12.9%)	2 (5.4%)	5 (13.9%)	3 (20.0%)	
	None	10 (6.5%)	2 (3.2%)	6 (16.2%)	2 (5.6%)	0 (0.0%)	

G1 (Western European), G2 (Middle Eastern), G3 (Latin American), G4 (Eastern European) countries’ participants

§Fisher’s Exact test. *Significant

On the other hand, most of participants (*n*=116, 75.3%) did not resort to *face mask fitting tests* before choosing their mask/respirator, with no significant differences between groups. The majority of participants (*n*=115, 74.4%) adopted the *“extended use” of masks*, and changed it only if soiled or damp. Only (*n*=22, 14.3%) immediately were replacing their masks after dealing with each patient, whether it became soiled or damp or not, while (*n*=17, 11%) adopted “extended use” and did not change it during the day even if soiled or damp. Statistically significant differences between groups are shown in Table 4.

For eye protection while dealing with patients, most of participants (*n*=89, 57.8%) commonly used a face shield/visor (that covers the front and sides of the face) with or without corrective glasses underneath. Other methods of eye protection along with the statistically significant differences between groups are represented in Table 4.

In terms of glove use, most of participants (*n*=111, 72.1%) always put on clean, non-sterile gloves before dealing with each patient, while 35 (22.7%) put on gloves only during an AGP, 4 (2.6%) put on gloves only whenever available, and 4 (2.6%) never used gloves and consider them non-essential. Most of participants (*n*=146, 94.8%) changed gloves immediately after dealing with each patient, whether or not they became torn or heavily contaminated, only 4 (2.6%) used the same gloves for as long as possible or throughout the day, and changed them only if torn or heavily contaminated, and none kept using them even if torn or soiled. No significant differences between groups are found. No statistically significant differences between groups were detected.

In terms of gown use, participants always put on a clean gown before dealing with each patient (*n*=60, 39%), only during an AGP (*n*=52, 33.8%), whenever available (*n*=24, 15.6%), while 18 (11.7%) participants never used gowns and considered them non-essential. Most of participants either changed gowns immediately after dealing with each patient, whether or not it is soiled (*n*=68, 44.2%) or used the same gown throughout the day and changed it only if soiled (*n*=64, 41.6%) and 4 (2.6%) used the same gown throughout the day and did not change it even if soiled. No statistically significant differences between groups were detected.

#### Adherence to adequate PPE use and airborne precautions during examinations/procedures that are potentially AGPs

Participants were first questioned if they were routinely performing specific procedures i.e., they were exposed to a higher risk of infection unless adequately protected. Those who stated that they do were further asked if they stuck to full PPE during that particular procedure. Less than half of participants (*n*=70, 45.5%) performed assessment and management of laryngectomy and tracheostomies e.g., voice restoration using voice prosthesis, cuff inflation or deflation, dysphagia management and stoma care, and all of them (*n*=70/70, 100%) adhered to full PPE during this. Most participants executed (*n*=105, 68.2%) and stuck to complete PPE (*n*=92/105, 87.6%) during Flexible Endoscopic evaluation of Swallowing (FEES). Likewise, most participants performed (*n*=127, 82.5%) and stuck to full PPE (*n*=111/127, 87.4%) during the trans-oral laryngeal examination with a rigid endoscope. Most participants performed (*n*=132, 82.5%) and adhered to complete PPE (*n*=113/132, 85.7%) during trans-nasal laryngeal or velopharyngeal examination with a flexible endoscope. Office-based injections into the larynx were performed by 56 (36.4%) of which 48/56 (85.7%) stuck to complete PPE during trans-oral and trans-nasal injections and 45/56 (80.4%) used full PPE during transcutaneous injections.

Trans-oral or trans- nasal esophagoscopy was performed by (*n*=49, 31.8%) and the majority (*n*=41/49, 83.7%) used complete PPE. Rehabilitative and compensatory approaches for dysphagia was carried out by (*n*=59, 38.3%) and the majority of those (*n*=46/59, 78%) reported that they stuck to full PPE during these approaches. Less than half of participants carried out (*n*=72, 46.8%) instrumental aerodynamic function assessment (microphones, face masks and intra-oral tubes) and the majority of those (*n*=55/72, 76.4%) stuck to PPE during this assessment with significant differences between groups (Table 5). Most participants performed (*n*=131, 85%) and used full PPE (*n*=97/131, 74%) during intraoral and oromotor examination. Bedside swallowing assessment was done by (*n*=66, 42.9%) and the majority of those (*n*=47/66, 71.2%) adhered to full PPE. Videofluroscopic Swallow Study (VFSS) was conducted by (*n*=41, 26.6%) and of those (*n*=29/41, 70.7%) adhered to complete PPE. Water swallow screening tests were carried out by (*n*=58, 37.7%) and the majority of them (*n*= 40/58, 69%) tended to follow full PPE. Most of participants performed (*n*=112, 72.7%) and stuck to complete PPE (*n*=73/112, 65.2%) during loud voicing/singing assessment tasks, with significant differences between groups (see Table 5).

**Table 5:** Participants’ adherence to personal protective equipment (PPE) use during examinations and procedures

	All sample (***N***=154)	G1 (***N***=62)	G2 (***N***=37)	G3 (***N***=36)	G4 (***N***=15)	***P***-value
**PPE use during loud voicing/singing tasks:**	73/112 (65.2%)	33/53 (62.3%)	10/19 (52.6%)	24/28 (85.7%)	5/11 (45.5%)	#0.036*
**PPE use during instrumental aerodynamic assessment:**	55/72 (76.4%)	28/33 (84.8%)	7/12 (58.3%)	16/18 (88.9%)	4/8 (50.0%)	§0.039*

G1 (Western European), G2 (Middle Eastern), G3 (Latin American), G4 (Eastern European) countries’ participants

# Chi-square test. §Fisher’s Exact test. *Significant

## Discussion

Caring for patients whilst securing their safety and ours is a true challenge during this unprecedented worldwide health crisis. This survey was conducted in the period when most countries were striving to flatten the curve of the first wave of the COVID-19 pandemic. As we find ourselves in the subsequent waves, meticulously looking at how we, as phoniatricians and otolaryngologists, complied with protective gear and infection control precautions, would help in analyzing and comparing our practice safety across the pandemic timeline.

PPE had been available for the great majority of participants in the different groups participating in this study, but for only half of Egyptian participants’ group. Similarly, only half of participants surveyed in an Egyptian study [16] were satisfied with the availability of PPE in their hospitals, and two thirds of participants had to personally purchase PPE. This may explain why participants from different groups in our study used PPE during AGPs comparably, despite variable official PPE supply.

WHO reported no PPE shortage in healthcare facilities in Egypt [17]. Contrary to WHO findings, Egyptian health care workers (HCWs) felt that PPE unavailability was largely (83.6%) responsible for their susceptibility to COVID-19 infection [18]. The National COVID-19 Response Plan in Egypt covered essential COVID-19 supplies, nevertheless, timely procurement was not always ensured due to funding issues [17]. Additionally, a shortage of PPE supplies in healthcare facilities was reported by 64.2% of those surveyed in low -income countries (versus 27.4% in higher- income countries) [19]. Such findings should stimulate health authorities to work towards increasing provision of protective gear inside hospitals, regardless of overall country income status, and this is a data-set that we will be investigating in follow up surveys.

Spread of disease is likely enhanced by HCWs’ lack of awareness of recommended infection control practices [20]. Attendance of infection control training courses is a significant factor that affected infection control practices [21]. Insufficient training on PPE use was viewed as a barrier to infection control practice by 87% of physicians in one study [21] and as a cause of more susceptibility to COVID-19 infection by one third of HCWs in another study [18]. Our survey demonstrated that most participants in the different groups had undertaken training on correctly using PPE, however less than half of Egyptian participants’ group were trained in this. Similarly, only around half of physicians surveyed in Egypt during the first wave had received infection control training and reported knowing the correct donning/doffing sequence [21]. Our survey findings were substantiated by a WHO fact-finding mission, which also reported that HCWs in Egypt were not properly trained to use PPE [17].

Only half of participants had received training on disinfecting tools and surrounds. Most likely this is due to a generally held conception that such tasks are usually handled by other staff, rather than physicians. Nonetheless, over two thirds of participants paid greater attention to disinfecting reusable equipment. Training in remedial actions (and how to deal with exposure) was found to be the most deficient (albeit to a lesser extent among Brazilian participants) type of infection control training among participants, with Egyptian participants significantly less trained. Moreover, approximately a quarter of all participants (again especially Egyptians) received no training at all. In April 2020, HCWs represented an alarming 13% of all COVID-19 cases in Egypt, according to the WHO (https://www.theguardian.com/global-development/2020/may/21/egypt-doctors-ppe-testing-coronavirus and https://dailynewsegypt.com/2020/04/13/13-of-egypts-coronavirus-infections-amongst-healthcare-workers-who/) compared to an average of 7% worldwide [6]. This finding underscores the importance of education as the key to mitigate the risk of viral transmission [5, 7, 9, 11, 19].

Over four fifths of participants ‘took the extra step’ and provisionally followed TBPs by considering every patient to be potentially infected. The most common TBP pattern followed was that of ensuring droplet precautions and moving to airborne precautions during all procedures. This complies with recommended guidance [5, 6]. Interestingly, none of the Egyptian participants followed airborne precautions at all times, and a greater percentage than in other groups followed SICPs alone (rather than TBPs) at all times, likely due to scarcity of required PPE or insufficient training.

HH is a primary determinant of the incidence of cross- transmission of nosocomial infections [22]. Adherence of the majority of participants to HH practice was noted in all situations related to patient care, including before and after all patient contact, after exposure to potentially infectious materials or touching patient’s surroundings. A lower degree of adherence to HH was noted before and after touching or readjusting face masks or handling of PPE, despite the available recommendation [12, 15]. This can be explained by insufficient training on the proper sequence of PPE donning and doffing, including HH steps.

Egyptian participants were the least likely of the various groups to perform HH before patient contact, again probably related to lack of training, while eastern European participants were the least likely to perform HH after exposure to infectious material and before or after adjusting masks (although they were not the least to receive training on infection control). The smaller number of participants in this subgroup might have skewed the results. These deficient aspects of HH practice should be highlighted in future education of phoniatricians and otolaryngologists.

Surgical masks loosely cover the nose and mouth, and are not generally reusable. They may protect against droplets but not aerosols [6, 7]. Filtering facepiece respirators e.g., FFP2/N95 and FFP3/N98 respirators are fluid-resistant, tight-fitting masks, filter both air inflow and outflow i.e., prevent two-way transmission, and are generally reusable when adequately sanitized, with little deterioration in efficacy. They protect against both droplet and airborne transmission [7, 9]. *Fit-testing* guarantees appropriate sizing, maintains a facial seal and protection against air leak [5, 6, 9, 10]. A PAPR should be used if available respirators fail to provide a complete seal on fit-testing. It actively circulates and filters air around the face, and frequently requires a surgical mask or an N95 respirator underneath [6, 7].

All participants used masks, with N95 being the most widely used mask type in western European and Brazilian participants while surgical masks were more commonly used by Egyptian participants. Deferral of use of N95 masks may not be solely related to PPE unavailability for this group but to inadequate knowledge as well, as their responses indicated that they recognized surgical masks as sufficient for their protection. Likewise, in an Egyptian study [21], most of surveyed physicians believed that all types of face masks have the same protection level against the infection, while only 3.8% believed they are not.

In order to avoid self-contamination and to save PPE resources, clinicians were recommended to consider using reusable N95 masks with periodic decontamination [7, 9, 15]. Most participants adopted *extended use of masks* and only changed them when soiled or damp, regardless of the PPE availability status within each group. It is also a consideration that a number of participants did not change these masks even if soiled or damp. On the other hand, participants who tended to change masks immediately after each use were the ones who used more surgical masks which are typically non-reusable.

Eye protection should be used whenever contamination to the eyes/face is likely as in AGPs [7, 9]. Face shields/visors do not substitute for masks [5, 9, 11]. They block initial forward air movement, but aerosols can move around them and disperse over wide areas. Additionally, hazardous downward jets can occur if the wearer is in a more elevated position [9]. The WHO suggestion of using face shields instead of masks, should only be undertaken in the context of severe mask shortages [5]. Corrective/medical eyeglasses and surgical loupes do not adequately protect from droplets, while safety glasses with/without side protections may provide more respiratory protection and occlusive eyewear like swim goggles offer even better protection [6, 7].

A face shield was by far the most widely used eye protection in all groups, followed by other measures. Nevertheless, nearly one fifth of participants either did not use any eye protection or used medical glasses only which are believed not to offer adequate protection [7].

Wearing gloves, a part of both standard and contact precautions, limits the risk of virus dissemination to the patient’s environment, to other patients and for the protection of HCWs [12]. Using gloves does not replace HH practice. Extended use or sterilization and reuse of single-use medical gloves is discouraged [12].

In accordance with recommendations, over two thirds of participants always used gloves before each patient contact, while nearly one quarter did so only with AGPs. The vast majority practiced single glove use per patient, as per guidelines [12]. This adherence was lower for gown usage as nearly 40% of participants put on a gown before each patient contact and one third did so only with AGPs. This did not seem to relate to accessibility to PPE, as such practice was similar across groups, and also only 15.6% reported that they used gowns only when available. Guidance advocates the use of gowns that are clean, non-sterile, long-sleeved, impermeable (or combined with a waterproof plastic apron underneath) for both AGPs and non-AGPs [6, 13, 14], and both under and above the potentially shared non-sterile lead apron during VFSS followed by doffing both after the procedure [5]. Gown use recommendations should thus be emphasized and will be an important focus of the next survey.

AGPs are considered high risk procedures and should be limited to urgent cases. However, to ensure continuity of care for patients, such procedures may be indicated in the context of COVID-19 pandemic provided that adequate PPE is properly applied. Two thirds of all participants showed adherence to full PPE while performing different AGPs. Such adherence was 100% while caring for tracheostomies and laryngectomees, between 80 to 88% for endoscopies and injection procedures, and between 69 to 78% for bedside dysphagia assessment and management as well as VFSS. This lower utilization of full PPE during VFSS as compared to PAPR may be because the setting does not always require the presence of physicians in close proximity to patients. Yet, VFSS should be considered an AGP as it might trigger cough [23]. Participants in different groups responded differently as to their adherence to full PPE use during some assessments, possibly due to lack of sufficient awareness of the hazardousness of these tasks.

## Conclusion

This study, carried out during the first wave of the COVID-19 pandemic, carefully surveyed, and analyzed the practice of phoniatricians and ENT specialists in many countries in relation to their abidance with recommended infection control precautions including HH and PPE use. Adequate compliance could be noted in many aspects, while others were still in need of further recognition. This emphasized the significance of infection control training programs which were not always prevalent in the surveyed group. We feel that it will be very valuable in our follow- on survey, to observe how these behaviors changed with time.

## Data Availability

The datasets used and analyzed during the current study are available from the corresponding author on reasonable request.

## Abbreviations

AGPs: Aerosol generating procedures
COVID-19: Coronavirus disease 2019
ENT: Ear, Nose and Throat
FEES: Flexible Endoscopic Evaluation of Swallowing
HCWs: Health care workers
HH: Hand hygiene
N95/98: refers to Non-oil, 95 or 98% efficiency, equivalent to FFP2/3: Filtering face piece respectively with the number indicating the level of protection
PAPR: Powered air-purifying respirator
PPE: Personal protective equipment
SARS-CoV2: Severe acute respiratory syndrome Coronavirus 2
SICPs: Standard infection control precautions
SPSS: Statistical Package for Social Sciences
TBPs: Transmission-based precautions (TBPs)
UEP: Union of European Phoniatricians
VFSS: Videofluroscopic Swallow Study
WHO: World Health Organization
